# PYCNOIB: Biodiversity and Biogeography of Iberian Pycnogonids

**DOI:** 10.1371/journal.pone.0120818

**Published:** 2015-03-17

**Authors:** Anna Soler-Membrives, Tomás Munilla

**Affiliations:** Unitat de Zoologia (BABVE), Universitat Autònoma de Barcelona, Cerdanyola del Vallès, Barcelona, Spain; University of Sydney, AUSTRALIA

## Abstract

Biodiversity and biogeographic studies comparing the distribution patterns of benthic marine organisms across the Iberian Atlantic and Mediterranean waters are scarce. The Pycnogonida (sea spiders) are a clear example of both endemicity and diversity, and are considered a key taxon to study and monitor biogeographic and biodiversity patterns. This is the first review that compiles data about abundance and diversity of Iberian pycnogonids and examines their biogeographic patterns and bathymetric constraints using GIS tools. A total of 17762 pycnogonid records from 343 localities were analyzed and were found to contain 65 species, 21 genera and 12 families. *Achelia echinata* and *Ammothella longipes* (family Acheliidae) were the most abundant comprising ~80% of the total records. The Acheliidae is also the most speciose in Iberian waters with 15 species. In contrast, the family Nymphonidae has 7 species but is significantly less abundant (<1% of the total records) than Acheliidae. Species accumulation curves indicate that further sampling would increase the number of Iberian species records. Current sampling effort suggests that the pycnogonid fauna of the Mediterranean region may be richer than that of the Atlantic. The Strait of Gibraltar and the Alboran Sea are recognized as species-rich areas that act as buffer zones between the Atlantic and Mediterranean boundaries. The deep waters surrounding the Iberian Peninsula are poorly surveyed, with only 15% of the sampling sites located below 1000 m. Further deep-water sampling is needed mainly on the Iberian Mediterranean side.

## Introduction

Biodiversity and biogeographic patterns of marine organisms in the Mediterranean and NE Atlantic are a relatively recent topic of study. There are still very few check-lists of Iberian marine invertebrate taxa, and studies comparing the distribution patterns of benthic organisms between Atlantic and Mediterranean waters around the Iberian Peninsula are still uncommon. Only a few studies on hydroids [1], peracarids [2], sponges [3,4] and ascidians [5] have compared the faunal assemblages between both sides of the Peninsula but these focused on the Strait of Gibraltar. Most studies did not find any clear differentiation along the Mediterranean-Atlantic interface leading to the conclusion that this is an homogeneous area [2] and that the major component of sublittoral Mediterranean fauna is of Lusitanian origin [4]. The Western Mediterranean basin has been identified as a marine biodiversity hot spot, characterized by high species endemicity [6,7].

The Pycnogonida (sea spiders) are one of the most intriguing groups of arthropods. These exclusively marine animals range from coastal shorelines to abyssal depths. They are distributed worldwide, with 1349 species and 77 genera described to date [8]. They have been used to study biogeographic patterns, mainly in the Southern Ocean [9,10].

Iberian pycnogonids have been studied since the beginning of the 20^th^ century. Studies are scattered throughout the region but have focused mainly on the Iberian Mediterranean, especially the Catalan Coast [11,12,13,14,15], the Alboran Sea [16,17,18,19], and Balearic Islands [20,21,22]. In contrast there have been relatively few studies on the pycnogonid fauna of the Iberian Atlantic coast. The principal works are those on the faunas of Portugal [23,24], northern Spain [22,25] and particularly on the Bay of Biscay [26]. Even fewer studies have been carried out within the Gibraltar zone [17,27,28] despite the known uniqueness of this region to study faunal assemblages. Of particular note is the BALGIM project, which focused on a study of the faunal transition between the Atlantic and the Mediterranean [28]. The project also addressed the correlation between the origin of the water masses flowing through the Strait of Gibraltar and the benthic faunas including the analysis of pycnogonids [28].

Prior to the present review, fifty-one pycnogonid species have been recorded from Iberian waters; 41 from the Atlantic Ocean and 30 from the Mediterranean Sea, with many common in both regions. Most species were collected during shallow water diving expeditions in the Mediterranean Sea. Specimens were commonly collected from macroalgae and phanerogams [12,15,29].

This is the first review of the pycnogonid fauna of the Iberian Peninsula to include the Mediterranean, the Atlantic watershed, and the Strait of Gibraltar transition zone. The main objectives of this study were 1) to provide the first complete review of the Iberian pycnogonid species, including distribution and bathymetric ranges, 2) to analyze species abundances, numbers of species and sampling intensity across the Iberian Atlantic-Mediterranean region, 3) to compare the biogeographic patterns of pycnogonids between the Atlantic and the Mediterranean Sea, 4) to discuss the role of the Strait of Gibraltar in influencing those patterns, and 5) to discuss bathymetric constraints on species range distributions. Evaluating the underlying forces that determine pycnogonid species diversity and distribution will further our greater understanding of the general biogeography of marine organisms across the north-east Atlantic and Mediterranean Sea.

## Materials and Methods

All available data on the occurrence of pycnogonids in the Iberian waters (including the area around the Balearic Islands) were gathered during the “Fauna Ibérica. Chelicerata. Pycnogonida” project (CGL2007–66786-C08–04). Data compilation used in the present study included all available published datasets (32 research publications), in addition to various unpublished records based on distinct survey collections including: the Museum Bocage (Portugal), DIVA-Artabria (Galician surrounding waters), INSUB (San Sebastián), El Cachucho (Asturias), Dragonera (Balearic Island), and the collections derived during the PhD research of both authors. References published and/or new data records for each species have been detailed in S1 Table. All these data have been now compiled and georeferenced into the Pycnoib-Database using MS Access. The locality of each sampling station is identified by latitude and longitude. Depth was included when available in the literature or collection reports.

### Species records and numbers of species

**Fig 1:**
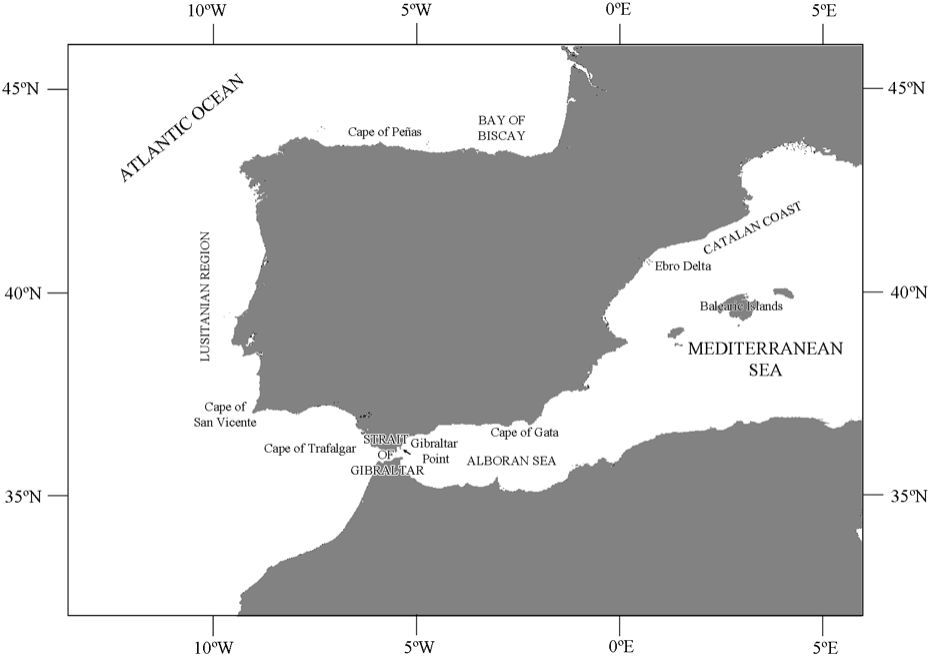
Map of the Iberian Peninsula and surrounding areas. The main topographical points which delimit some small geographical areas are shown.

The study area (Fig. 1) was divided into a series of 160 grid cells of 0.3 degrees of latitude and 0.3 degrees of longitude, covering three main regions, the Atlantic, the Mediterranean and the Strait of Gibraltar. The selected grid cell size provides a proper display of the biogeographic patterns without diminishing the spatial accuracy (see further discussion on S1 Information). Although the International Hydrographic Organization defines the limits of the Strait of Gibraltar by the Trafalgar Cap in the west and the Europa Point (Gibraltar Point) in the east, in this analysis the limits are defined as being from 6°30’00”W on the west side to 5°00’00”W (Estepona) on the east side. For further detail, some specific areas within the Atlantic and Mediterranean regions were defined, such as the Bay of Biscay, the Atlantic Portuguese (Lusitanian area), the Alboran Sea and the Catalan coast (from the Ebro Delta northwards to the Spanish boundary). The definition of these areas is based upon maritime areas such as seas or archipelagos, and the main factors influencing marine organisms, such as currents or eddies (see S2 Table for further details).

The number of specimens was counted to determine the abundance (N) and species were identified to determine the number of species (S) for each genus and family. The number of distinct species for each grid cell was also calculated. The species accumulation curves (SAC) were used to predict total species numbers for each study area and region and were calculated using the species-accumulation plot option in PRIMER v5 software [30], with 999 permutations.

### Distribution and bathymetric patterns

The geographic distribution of each species was mapped using a geographic information system, GIS (ARCGIS 10.0 program, ESRI, Redlands, CA). Maps were constructed based on data from the earliest record [31] to the most recent record and are, therefore, cumulative rather than current distributions maps.

Species with similar distribution ranges were grouped together into a set of general distribution patterns with the number of species in each group recorded. Species found in a single grid cell or in at most three grid cells but within close proximity (2–3 grid cells in between) were identified as species exclusive to that area within the definition framework of Iberian waters. Species found in an area and which have no other global record were considered to be endemic.

In order to analyze biogeographic relationships amongst Iberian pycnogonids, and accounting for the different sampling regimes involved, a qualitative Bray-Curtis similarity analysis of presence/absence data was performed using the multivariate statistical software PRIMER [32]. The resulting similarity matrix was then used for cluster analysis. Only grid cells with three or more species present were used in this analysis. Cluster groupings have not been statistically tested, but are determined subjectively.

Minimum and maximum depths were recorded for each species based on available data and depth ranges were documented for each genus and family.

## Results

### Species records and numbers of species

**Fig 2:**
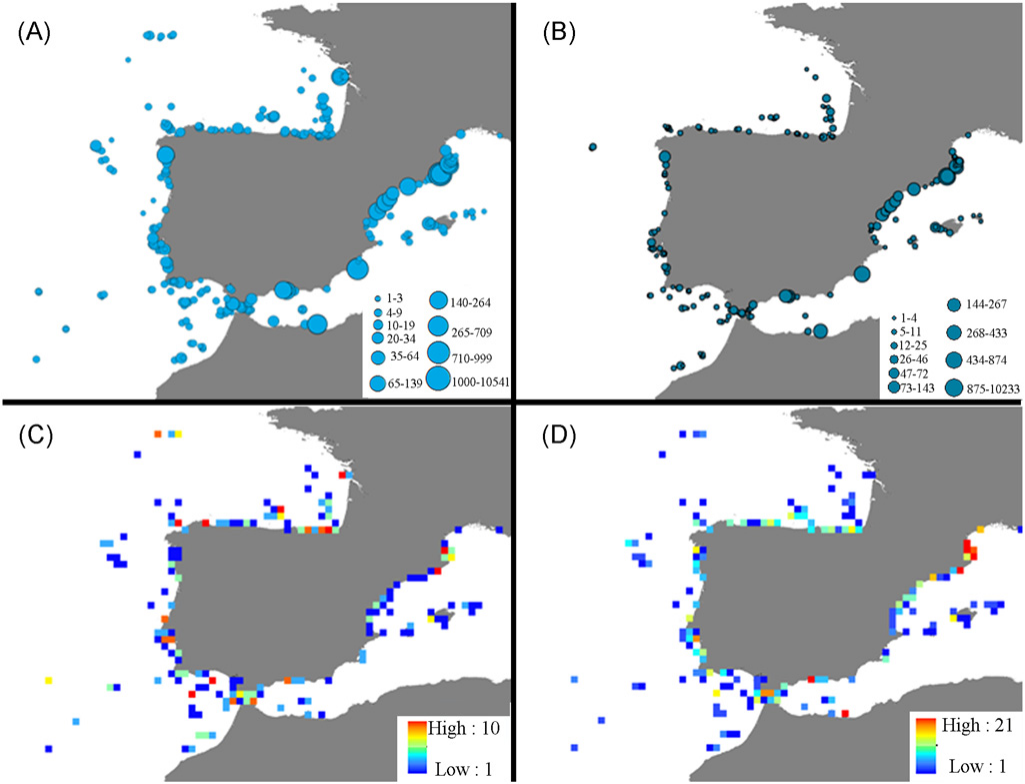
Quantifying the distribution of Iberian pycnogonids. Distribution of sample occurrences of Iberian pycnogonids (A) and specimens belonging to the most abundant family Acheliidae (B). Number of sampling sites of each 0.3° by 0.3° grid cell (C) and number of species from each 0.3° by 0.3° grid cell (D).

A total of 17762 pycnogonid records from 343 localities in the Iberian Atlantic-Mediterranean waters were compiled (Fig. 2A, Table 1). Only 27 of these records could not be geo-referenced and were subsequently excluded from the biogeographic analysis. In total, 65 species (14 of which were new records) belonging to 21 genera and 12 families were identified.

**Table 1:** Occurrences and numbers of species (S) by family and genus of Iberian pycnogonids, showing the percentage of contribution by family.

Family	Genus	Occurrences	% Occurrences	S	%S
Acheliidae		14166	79.75	15	23.08
	*Achelia*	5593	31.49	3	
	*Ammothella*	8335	46.93	7	
	*Cilunculus*	20	0.11	2	
	*Hannonia*	1	0.01	1	
	*Paranymphon*	190	1.07	1	
	*Neotrygaeus*	27	0.15	1	
Ascorhynchidae		98	0.55	6	9.23
	*Ascorhynchus*	94	0.53	5	
	*Nymphonella*	4	0.02	1	
Tanystylidae		1152	6.49	2	3.08
	*Tanystylum*	1152	6.49	2	
Nymphonidae		138	0.78	7	10.77
	*Nymphon*	138	0.78	7	
Callipellenidae		1040	5.86	6	9.23
	*Callipallene*	1040	5.86	6	
Pallenopsidae		8	0.05	3	4.62
	*Bathypallenopsis*	8	0.05	3	
Phoxichilidiidae		847	4.77	9	13.85
	*Anoplodactylus*	847	4.77	9	
Endeidae		118	0.66	2	3.08
	*Endeis*	118	0.66	2	
Colossendeidae		112	0.63	6	9.23
	*Colossendeis*	97	0.55	5	
	*Hedgpethia*	15	0.08	1	
Austrodecidae		19	0.11	2	3.08
	*Austrodecus*	3	0.02	1	
	*Pantopipetta*	16	0.09	1	
Rhynchotoraxidae		6	0.03	2	3.08
	*Rhynchothorax*	6	0.03	2	
Pycnogonidae		58	0.33	5	7.69
	*Pentapycnon*	3	0.02	1	
	*Pycnogonum*	55	0.31	4	
12	21	17762	100	65	100.00

**Fig 3:**
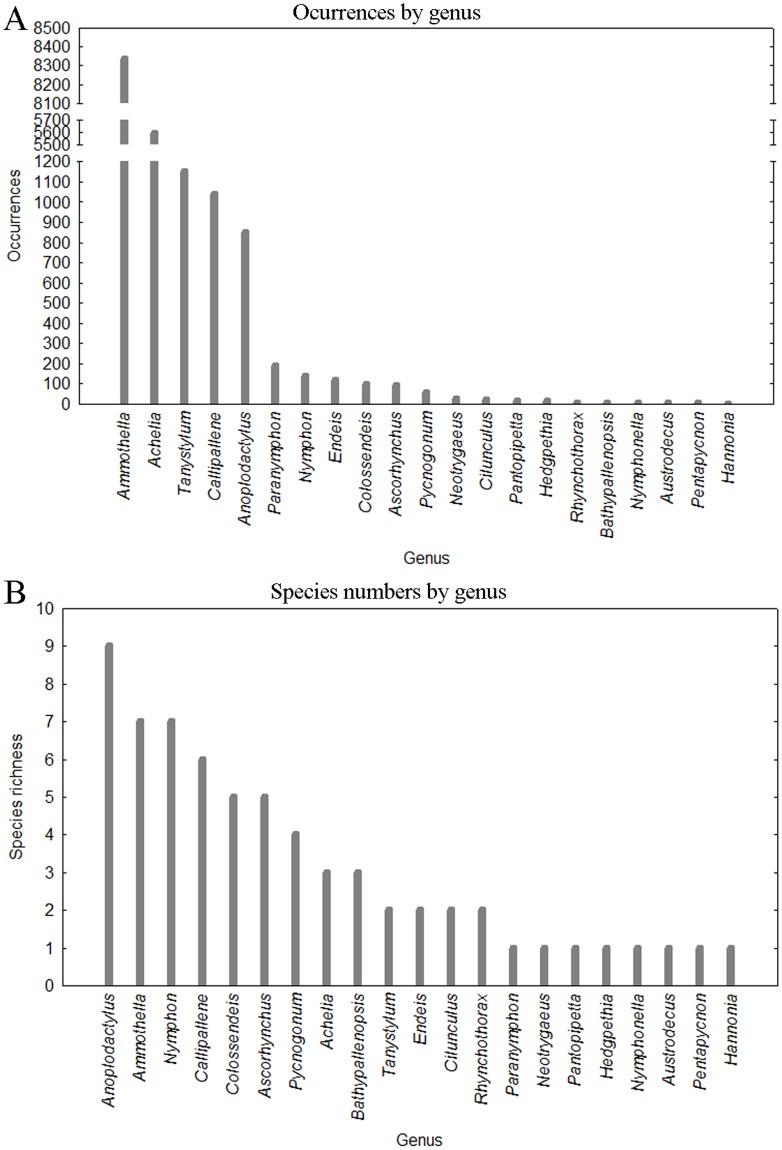
Occurrences (A) and numbers of species (B) of Iberian pycnogonids by genus.

Acheliidae is the most abundant (~80%) and diverse family. Most families represented less than 1% of the total abundance and are poorly diverse in Iberian waters (Table 1). The most species-rich genus is *Anoplodactlylus* (Fig. 3). Approximately 75% of the total abundance is dominated by two species *Ammothella longipes* and *Achelia echinata*, most being recorded from the Mediterranean shore (Table 2). While the genus *Achelia* is highly abundant throughout the Iberian waters (over 5000 specimens), it has low species diversity (3). In contrast, the genus *Nymphon* has low abundance (N = 138, that is <1%) but has high species diversity (7 species).

**Table 2:** Occurrences of the Iberian pycnogonid per region.

Species	N	% N	ATL	MED	GIB	Pattern	IP Distribution	Iberian depth	Species depth range	WW Distribution
*Achelia echinata* Hodge, 1864	5243	29.5	167	5006	70	A	Bis, NIb, Gal, Por, Gib, Alb, EMed, Bal, Cat	1–150	1–537	C
*Achelia langi* (Dohrn, 1881)	121	0.7	39	79	3	Other	Por, Gib, Alb, EMed, Cat	0–25	0–100	AM (EA, M)
*Achelia vulgaris* (Costa, 1861)	229	1.3	63	165	1	A	NIb, Gal, Por, Gib, Alb, EMed, Cat	0–62	0–400	AM (EA, M)
*Ammothella appendiculata* (Dohrn, 1881)	49	0.3		49		B	Alb, EMed, Cat	0–35	0–65	C
*Ammothella biunguiculata* (Dohrn, 1881)	11	0.1		10	1	Other	Gib, Cat	2–24	0–45	C
*Ammothella gibraltarensis* Munilla, 1993	1	0.0			1	D	Gib	76–80	76–80	E (Gib)
*Ammothella longioculata* (Faraggiana, 1940)	1	0.0		1		D	Alb	12	0–127	E (Med)
*Ammothella longipes* (Hodge, 1864)	7717	43.4	86	7598	33	A	Bis, NIb, Gal, Por, Gib, Alb, EMed, Bal, Cat	0–40	0–87	AM (EA, M)
*Ammothella tubicen* Stock, 1978	3	0.0	3			D	Bis	1980–1995	1980–1995	E (Bis)
*Ammothella uniunguiculata* (Dohrn, 1881)	553	3.1		552	1	B	Gib, Alb, EMed, Cat	0–22	**0(3)-22(16)**	E (Med)
*Anoplodactylus angulatus* (Dohrn, 1881)	86	0.5	25	55	6	A	Bis, NIb, Gib, Alb, Bal, Cat	0–44	0–45	AM (EA, M)
*Anoplodactylus arnaudae* Stock, 1978	13	0.1	12		1	C	Oatl, Gib	235–1350	165–1350	EA
*Anoplodactylus nanus* Krapp, Kocak & Kagatan, 2008	1	0.0		1		D	**Cat**	2–15	1–**15(2)**	**E (Med)**
*Anoplodactylus oculatus* Carpenter, 1905	1	0.0	1			C	OAtl	35–905	**35(560)-905(850)**	EA
*Anoplodactylus petiolatus* (Krøyer, 1844)	461	2.6	353	72	36	A	Bis, NIb, Gal, Por, OAtl, Gib, Alb, Cat	0–1200	0–1500	AM (TA, M)
*Anoplodactylus pygmaeus* (Hodge, 1864)	173	1.0	17	155	1	A	Bis, NIb, Gal, Por, Gib, Alb, EMed, Cat	0–2000	0–**2000(587)**	AM (TA, M)
*Anoplodactylus robustus* (Dohrn, 1881)	6	0.0		6		B	Alb, Cat	34	4–44	C
*Anoplodactylus typhlops* Sars, 1888	13	0.1	13			C	OAtl	569–1250	400–3620	C
*Anoplodactylus virescens* (Hodge, 1864)	93	0.5	74	13	6	A	Bis, NIb, Gal, Por, Gib, Alb, Cat	0–40	0–40	D (St. Paul I., Amsterdam, M)
*Ascorhynchus abyssi* Sars, 1877	1	0.0	1			C	OAtl	1500	900–4350	EA
*Ascorhynchus castelli* (Dohrn, 1881)	8	0.0	2	6		Other	Bis, Cat	1–25	0–130	AM (WA, M)
*Ascorhynchus pudicus* Stock, 1970	12	0.1	10		2	C	Oatl, Gib	150–320	120–360	AM (EA, WM)
*Ascorhynchus simile* Fage, 1942	15	0.1	11	4		Other	Bis, Gal, EMed, Cat	0–35	0–100 (1 record 1238 m)	AM (WA, WM)
*Ascorhynchus turritus* Stock, 1978	58	0.3	58			C	Bis, OAtl	1894–2813	1894–4411	TA
*Austrodecus conifer* Stock, 1991	3	0.0	3			C	OAtl	675–685	675–811	EA
*Bathypallenopsis juttingae* (Stock, 1964)	1	0.0	1			C	OAtl	400	325–1813	D (EA, New Caledonia)
*Bathypallenopsis longirostris* (Wilson, 1881)	3	0.0	3			C	Bis, OAtl	1200–2627	135–3550	C (No-M)
*Bathypallenopsis scoparia* (Fage, 1956)	4	0.0	2	1	1	Other	Bis, Gib, Bal	580–1200	400–1520	C **(WM)**
*Callipallene brevirostris* (Johnston, 1837)	15	0.1	2	13		Other	Gal, Por, Alb	0–170	0–316	AM (TA, M)
*Callipallene emaciata* (Dohrn, 1881)	461	2.6	137	301	23	Other	Gal, Por, Gib, Alb	0–362	0–**362(45)**	AM (TA, M)
*Callipallene phantoma* (Dohrn, 1881)	13	0.1	2	11		Other	Gal, Por, Alb, EMed, Bal, Cat	1–574	0–850	AM (TA, M)
*Callipallene producta* (Sars, 1881)	84	0.5	26	51	7	A	NIb, Gal, Por, OAtl, Gib, Alb, Cat	3–1360	3–1550	AM (EA, WM)
*Callipallene spectrum* (Dohrn, 1881)	114	0.6	1	108	5	Other	Gal, Gib, Alb, Bal, Cat	0–44	0–160	AM (EA, WM)
*Callipallene tiberi* (Dohrn, 1881)	353	2.0	7	330	16	A	Bis, Oatl, Gib, Alb, Cat	0–1360	0–**1360(523)**	AM (EA, WM)
*Cilunculus alcicornis* Stock, 1978	9	0.1	9			C	NIb, OAtl	569–1125	**569(650)**-1140	EA
*Cilunculus europaeus* Stock, 1978	11	0.1	11			C	NIb, OAtl	569–1200	463–1576	EA
*Colossendeis angusta* Sars, 1877	6	0.0	6			C	OAtl	1805–2579	12–5480	C
*Colossendeis arcuata* Milne-Edwards, 1885	4	0.0	4			C	OAtl	1747–2177	500–2220	C
*Colossendeis clavata*, Meinert, 1899	6	0.0	6			C	OAtl	1878–2282	994–3100	TA
*Colossendeis colossea* Wilson, 1881	20	0.1	20			C	NIb, Por,OAtl	906–2579	420–5200	C
*Colossendeis macerrima* Wilson, 1881	61	0.3	61			C	NIb, OAtl	1747–4411	121–4411	C
*Endeis charybdaea* (Dohrn, 1881)	7	0.0	2	5		Other	Bis, NIb, Cat	10–210	**10(15)**-250 (1 record 800 m)	AM (EA, M)
*Endeis spinosa* (Montagu, 18808)	111	0.6	46	60	5	A	Bis, NIb, Gal, Por, Gib, Alb, EMed, Bal, Cat	0–44	0–100 (1 record 537 m)	AM (TA, M)
*Hannonia stocki* Munilla, 1993	1	0.0			1	D	Gib	76–80	76–80	E (Gib)
*Hedgpethia atlantica* (Stock, 1970)	15	0.1	13		2	C	OAtl, Gib	135–1125	100–1125	EA
*Nymphon caldarium* Stock, 1987	6	0.0			6	D	Gib	340–580	340–580	E (Gib)
*Nymphon gracile* Leach, 1814	82	0.5	65	16	1	A	Bis, NIb, Gal, Por, Gib, EMed, Cat	0–40	0–52	AM (EA, M)
*Nymphon laterospinum* Stock, 1963	24	0.1	24			C	NIb, OAtl	1894–4715	1890–4715	TA
*Nymphon macrum* Wilson, 1880	1	0.0	1			D	Por	78	35–1500	TA
*Nymphon puellula* Krapp, 1973	3	0.0			3	D	**Gib**	32	32–35	E(WM)
*Nymphon tricuspidatus* Soler-Membrives & Munilla, 2011	21	0.1	21			C	Bis, Gal	569–993	569–993	EA
*Nymphon tubiferum* Stock, 1978	1	0.0	1			D	Por	740	740	EA
*Nymphonella tapetis* Ohshima, 1927	4	0.0		4		D	Cat	2–4	0–15	D (Japan, WM)
*Pantopipetta armoricana* Stock, 1978	16	0.1	16			C	Gal, OAtl	180–1000	**180(200)**-1210	EA
*Paranymphon spinosum* Caullery, 1896	190	1.1	188	2		Other	Bis, NIb, Gal, Por, OAtl, Cat	60–2076	67–2300	C
*Pentapycnon geayi* Bouvier, 1911	3	0.0			3	D	Gib	12–42	8–70	TA
*Pycnogonum litorale* (Strom, 1762)	14	0.1	14			C	Bis, NIb, Por, OAtl	150–400	0–1262	AM (TA, M)
*Pycnogonum nodulosum* Dohrn, 1881	29	0.2		29		B	Alb, Bal, Cat	0–44	0–49	AM (EA, M)
*Pycnogonum plumipes* Stock, 1960	4	0.0		4		D	Cat	10–30	1–126	E(WM)
*Pycnogonum pusillum* Dohrn, 1881	8	0.0		8		B	Alb, Cat	3–24	0–35	AM (EA, M)
*Rhynchothorax mediterraneus* Costa, 1861	5	0.0		2	3	B	Gib, Bal	55–135	1–200	C
*Rhynchothorax voxorinus* Stock, 1966	1	0.0		1		D	Cat	10–18	10–18	E (Med)
*Tanystylum conirostre* (Dohrn, 1881)	1092	6.1	14	1063	15	A	Bis, NIb, Por, Gib, Alb, EMed, Bal, Cat	0–490	0–**490(45)**	AM (TA, M)
*Tanystylum orbiculare* Wilson, 1878	60	0.3	4	56		Other	Por, Alb, EMed, Bal, Cat	0–2028	0–**2028(60)**	AM (TA, M)
*Neotrygaeus communis* Dohrn, 1881	27	0.2		27		B	Alb, EMed, Bal, Cat	0–24	0–24	E (Med)
**N**	**17762**	**100**	**1645**	**15864**	**253**					
**S**	**65**		**47**	**36**	**27**					

**Fig 4:**
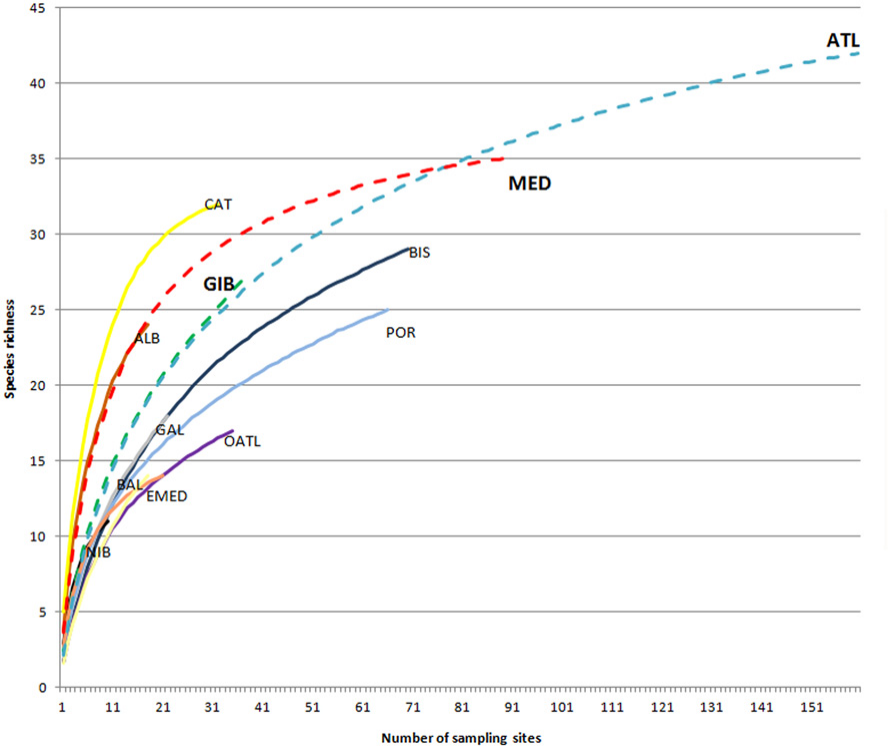
Species accumulation curves showing accumulation of pycnogonid species by area and region. Areas (solid lines): BIS, Bay of Biscay; NIB, north Iberian Peninsula; GAL, Galician waters; POR, Portugal; OATL, Atlantic open-ocean; ALB, Alboran Sea; EMED, eastern Iberian Mediterranean Sea; BAL: Balearic Islands; CAT, Catalan coast. Regions (dashed lines): GIB, Strait of Gibraltar; ATL, Iberian Atlantic region; MED, Iberian Mediterranean side.

**Fig 5:**
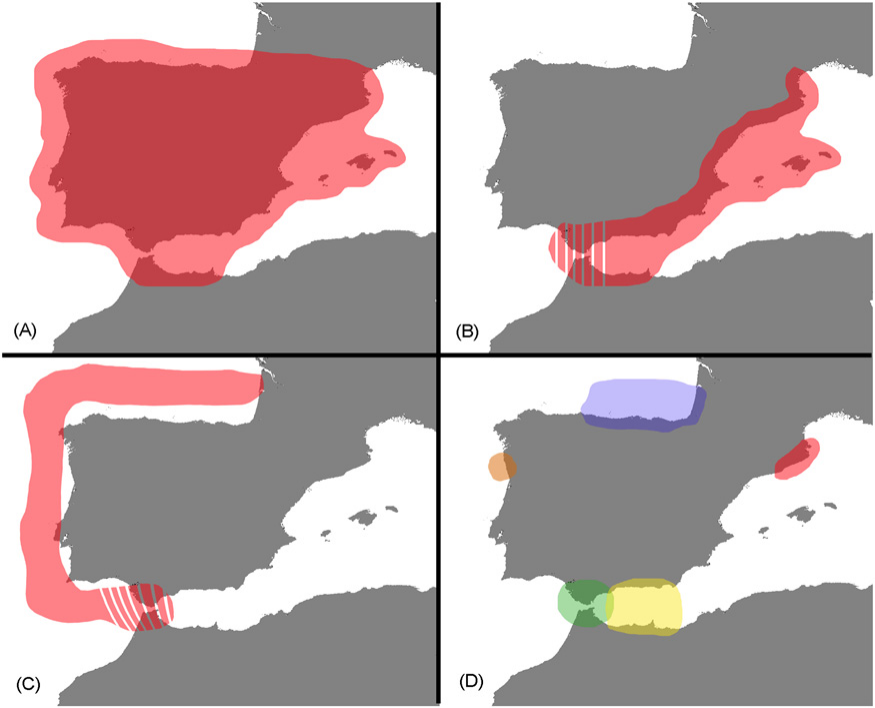
Major distribution patterns of pycnogonid species. The number of species per patterns are: A = 12, B = 7 (2 including the Strait of Gibraltar), C = 21 (3 including the Strait of Gibraltar) and D = 13 (5 exclusive to the Strait of Gibraltar, 4 to the Catalan coast, 2 to Portugal, and 1 to each Bay of Biscay and Alboran Sea).

Patterns and codes of Iberian distribution are related to Figs. 4 and 5. Bathymetric ranges of Iberian pycnogonid species are also shown, as well as their worldwide bathymetric and geographic distributions (C, cosmopolitan; AM, Atlantic-Mediterranean; TA, trans-Atlantic; EA, eastern Atlantic Ocean; WA, western Atlantic Ocean; M, Mediterranean Sea including eastern and western basins; WM, western Mediterranean basin; E, endemic; D, disrupted distribution). In bold, new distribution ranges not published before, and between brackets previous bathymetric distributions before these new data.

Pycnogonid records are unevenly distributed and largely reflect the uncoordinated sampling efforts (Fig. 2C). Sampling intensity within each of the 0.3° by 0.3° grid cells is generally low. Only a few areas around the Costa Brava (Catalonia), Gibraltar zone and the Bay of Biscay are well sampled. In contrast, large areas along the Atlantic Portuguese coastline, the eastern Mediterranean coast (EMed) and the Balearic Islands are poorly sampled. Most of the sampling sites are restricted to shallow coastal waters, while the deep-sea areas of the Iberian Mediterranean and Atlantic remain unsampled.

Species numbers are also unevenly distributed among grid cells (Fig. 2D) but interestingly, do not always correlate with sampling efforts within those areas. For example, despite high sampling intensity in a number of grid cells within the Bay of Biscay and at deep-sea stations to the far northwest (Fig. 2C), species numbers in those localities are low (1–2 species identified, Fig. 2D). In other areas such as, the Costa Brava, which is one of the best sampled zones (Fig. 2C), species numbers are relatively high (21 species found).

The species accumulation curves of the areas studied show, not unexpectedly, that increased sampling efforts generally lead to increase the number of species (Fig. 4). Moreover, many of the curves do not reach their asymptote, suggesting that the number of species in those areas may increase, even in relatively well-sampled areas. The accumulation curve for the Catalan coast levels off, suggesting the majority of the pycnogonid diversity is well described in this area. When comparing species counts at a fixed number of sample sites, a high degree of species accumulation is shown by the Alboran curve, although it does not reach the asymptote. This pattern is followed by the Gibraltar zone, with a little lower degree of slope. Atlantic regions (open Atlantic stations, Portuguese waters and Bay of Biscay) show the lowest species numbers by sampling site (Fig. 4).

### Biogeographic patterns of Iberian pycnogonids

Comparisons between the Atlantic and Mediterranean sides of the Iberian Peninsula (excluding the Strait of Gibraltar), reveal a number of interesting differences in numbers of species and abundances. Absolute number of species in the Iberian Atlantic is greater than in the Mediterranean whereas species abundances in the Atlantic are much lower than in the Mediterranean. For example, 47 pycnogonid species have been identified in the Iberian Atlantic with 24 of them exclusive to these waters, whereas in the Iberian Mediterranean only 36 species have been identified with 13 exclusive to this region. The difference in the number of species between the two is most likely correlated with the differences in sampling efforts with 234 localities surveyed in the Atlantic compared with only 87 sites in the Mediterranean. Therefore, the Iberian Atlantic number of species per station is half (S/St = 0.2) compared to the Iberian Mediterranean ratio (S/St = 0.4). However, despite the greater number of sampling sites and species diversity found in the Iberian Atlantic waters, species abundances are nearly an order of magnitude lower than in the Mediterranean (1645 Atlantic specimens versus 15864 Mediterranean specimens).

Rarefaction analysis (Fig. 4) shows that the Mediterranean region has a steeper slope than the Atlantic region but tends to plateau off around 35 species. The Atlantic region does not reach asymptote as early (<45 species), indicating a lower level of local species numbers but a higher level of regional species numbers. The Gibraltar curve follows the slope of the Atlantic region curve. Instead, the adjacent Alboran Sea follows the Mediterranean pattern, with higher levels of species counts than the Strait of Gibraltar. The Catalan coast has the steepest slope indicating particularly high species numbers (around 30 species for 20 sampling locations). The Iberian Atlantic areas (mainly Bay of Biscay, Portuguese coast and open Atlantic waters) tend to have low levels of species numbers per sampling site compared to those from the Mediterranean.

The geographical distribution pattern of each species is detailed in Table 2 and Fig. 5. The most geographically widespread distribution pattern (shown by 12 species) is that of the Atlantic-Mediterranean, which includes all of the waters surrounding the Iberian Peninsula (pattern A, Fig. 5A). Species are considered to have this pattern if they have been recorded from at least two areas within each the Atlantic and the Mediterranean regions, and have also been recorded in the Strait of Gibraltar. The Mediterranean pattern (including the Strait of Gibraltar but absent from the Atlantic, pattern B, Fig. 5B) is found in seven species with only two species (*Ammothella uniunguiculata* and *Rhynchothorax mediterraneus*) occurring in the Strait of Gibraltar. The Atlantic pattern (including the Strait of Gibraltar but absent from the Mediterranean, pattern C, Fig. 5C) is by far the most common being found in 21 species, of which again only two (*Ascorhynchus pudicus* and *Hedgpethia atlantica*) are also found in the Gibraltar area. Thirteen species are narrowly restricted to small, localized areas (pattern D, Fig. 5D); 5 are exclusive to the Strait of Gibraltar (3 of them endemic to this region), 4 are exclusive to the Catalan coast (though none are endemic), 2 are exclusive to the Portuguese Atlantic zone, one is exclusive to the Alboran Sea, and one is endemic to the Bay of Biscay. The remaining patterns, which are found in only 1–2 species, such as disrupted distribution in the Strait of Gibraltar and Catalan coast, and Atlantic-Mediterranean distribution excluding the north peninsular side, among others, are grouped under the category of “others” (Table 2).

**Fig 6:**
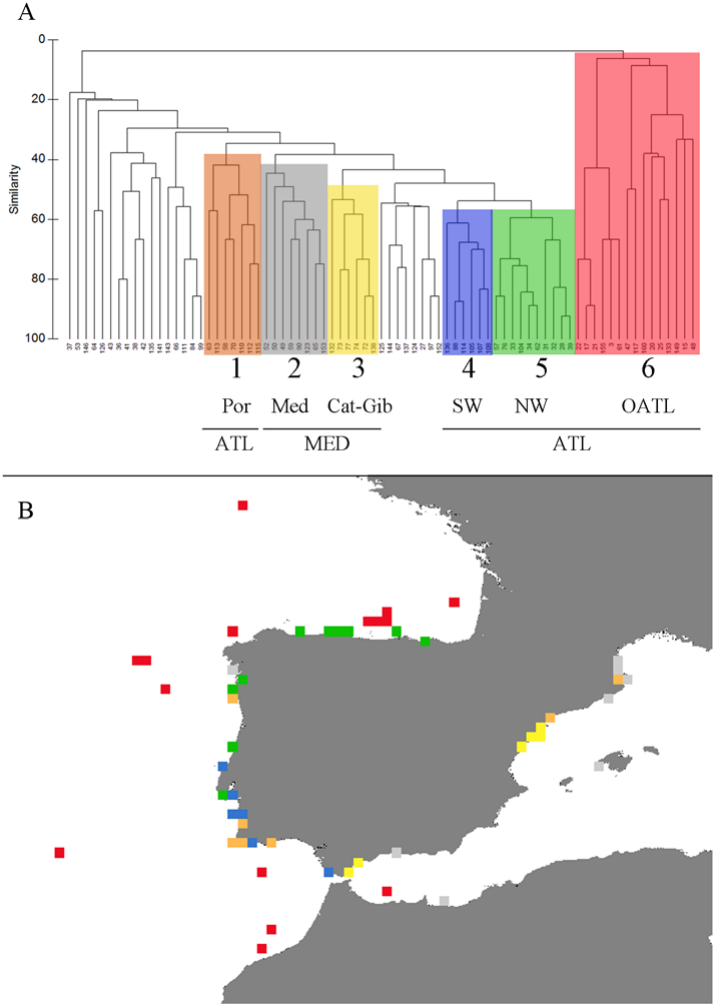
Biogeographic relationships in pycnogonid Iberian species assemblage in 0.3° by 0.3° grid cells. Cluster analysis based on the Bray-Curtis faunal similarity among 0.3° by 0.3° grid cells that contain three or more species of Iberian pycnogonids (A), and geographic representation of the cluster distribution.

The results of the Bray-Curtis similarity analysis based on the presence/absence data of all grid cells containing 3 or more species (Fig. 6A) were mapped onto their geographic locations (Fig. 6B). The clusters show six distinct groups separated into two main branches. The cluster 6 (red, open Atlantic, OAtl) is composed of all Atlantic deep-water grid cells, plus two Atlantic grid cells at depths around 200 m but which are located very far from the coast (see map Fig. 6B). The left branch is composed of all other groupings (1–5) plus grid cells not included in a group. This branch is composed of both Mediterranean and Atlantic stations regardless of depth, in agreement with the Mediterranean-Atlantic pattern. Cluster 1 (orange) is composed mostly of Portuguese stations (except from 58 and 70 grid cells on the Catalan coast). Clusters 2 (grey) and 3 (yellow) are exclusively Mediterranean stations, with the exception of one grid cell in Galician waters (n° 50). Cluster 3 agrees with the disrupted distribution of the Gibraltar region and Catalan coast. Cluster 4 (blue, SW) is formed by grid cells situated in the south-western corner of the Iberian Peninsula, i.e. south of Portugal and Gibraltar, and cluster 5 (green, NW) is comprised of grid cells situated in the north and western corner of the Iberian Peninsula, that is north of Portugal, north Spain, and one Biscayan grid cell. Clusters 4 and 5 are consistent with the Atlantic pattern (pattern C, Fig. 5C), and together with cluster 6, are exclusive to the Atlantic.

### Bathymetric distributions of the Iberian pycnogonids

**Fig 7:**
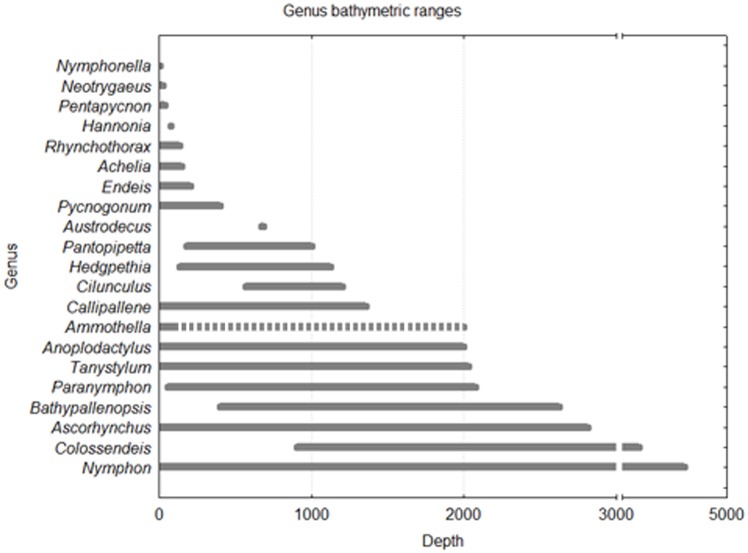
Depth ranges of Iberian pycnogonids by genus. Dashed line in *Ammothella* indicates disrupted bathymetric distribution (all species present at 0–80 m depth but *A*. *tubicen* at 1980–1995 m).

The inclusion of previously unpublished data from cruises and collections resulted in the increase of the bathymetric range of eleven species (Table 2). For example, the genus *Tanystylum*, represented in Iberian waters by two species, *T*. *conirostre* and *T*. *orbiculare*, had only been previously recorded from shallow waters (above 45–60 m). The present study has seen large increases in both species bathymetric ranges; up to 490 m depth for *T*. *conirostre* and up to 2028 m for *T*. *orbiculare*. The two most abundant genera in Iberian waters, *Achelia* and *Ammothella*, are restricted to very shallow waters, being found only above depths of 150 m and 80 m, respectively. The unique exception is *Ammothella tubicen*, which is encountered at depths around 2000 m (Table 2, Fig. 7). Some deep-sea specialists’ genera, e.g. the genus *Colossendeis*, are only found at depths greater than 900 m.

In terms of diversity, the exclusive shallow Iberian continental shelf pycnogonid fauna (depths shallower than 200 m) is composed by 7 genera (*Nymphonella*, *Neotrygaeus*, *Pentapycnon*, *Hannonia*, *Rhynchothorax*, *Achelia* and *Endeis*) and 31 species. Iberian pycnogonids found exclusively at depths below 200 m are represented by 20 species, but belonging to four genera (*Pantopipetta*, *Cilunculus*, *Bathypallenopsis* and *Colossendeis*). In terms of abundance, only 429 out of 17762 records occurred below the 200 m depth.

## Discussion

The Iberian region is particularly important to monitor changes in biogeographic distribution patterns, species diversities and abundances. As due to changing climatic conditions it is experiencing local species extinctions and increased colonization by invasive species [6]. Pycnogonids are a clear example of both endemicity and diversity [33,34]. Although they are among the better studied groups of marine invertebrates in areas such as the Southern Ocean [9,34,35], the biogeography and diversity of most temperate pycnogonids are poorly understood. This is the first review to focus on the diversity and distribution of the pycnogonid fauna from the Atlantic and Mediterranean Iberian Peninsula.

### Species records and numbers of species

The Iberian pycnogonid fauna (65 species) is more diverse compared to those in neighboring countries such as France (46 species [36,37]), Italy (45 species [38]) and the UK (37 species [39]). This higher Iberian diversity may be due to the converging of the two major different water masses, i.e. the almost enclosed Mediterranean Sea and the open Atlantic Ocean, with their different oceanographic and biological characteristics. Furthermore, the results of the SAC analysis (Fig. 4) indicates that further sampling would likely lead to the discovery of even greater numbers of Iberian pycnogonid species.

As with other taxonomic groups [6,40], the knowledge of pycnogonid distributions within the Iberian waters may be biased in favor of shallow zones located near to marine research institutions, which have traditionally been the focal sites of most scuba-diving surveys [40]. In comparison, there have been few long-range off-shore scientific surveys and this is evident in the patchy distribution of the more outlying and deep-water sampling localities (Fig. 2C). *Anoplodactylus nanus* was recorded for the first time in the western basin of the Mediterranean Sea (Costa Brava coast, NE Iberian Peninsula) and is only the second time it has been recorded globally [41]. The presence of *Nymphon puellula* in Ceuta (Strait of Gibraltar) is reported for the first time, and constitutes the second record of the species since being described in Catania (Sicilia, Italy) [42].

### Biogeographic patterns of Iberian pycnogonids

Even after taking into account the degree of sampling biases, there are still a number of notable biogeographic patterns. The species with the Atlantic-Mediterranean distribution pattern (Fig. 5A) are also the most abundant and frequently recorded, with one of the species (*Achelia echinata*) also being found globally (Table 2). It is the most widespread biogeographic distribution across the Iberian Peninsula and is therefore likely to be found in generalist species that have no restrictive environmental and ecological requirements (e.g. salinity, water temperature, habitat and substrate).

The greater abundance of pycnogonids found on the Iberian Mediterranean side could be attributed to the approximately 13000 specimens of three species collected from a single sampling locality (*A*. *echinata*, *A*. *longipes* and *T*. *conirostre*). However, when these records are omitted, the abundance of pycnogonids in the Mediterranean is still double that of the Atlantic. Species diversity per sampling station in the Mediterranean is also double that of the Atlantic despite total species numbers being higher in the Atlantic. The rarefaction curve technique is useful as it allows comparison between regions with different numbers of samples. Well-sampled Mediterranean cell grids (off the Catalan coast and in the Alboran Sea) have higher levels of species numbers compared to the well-sampled Atlantic areas (Biscayan and Galician coast) (Fig. 4). Therefore, the sublittoral pycnogonid fauna of the Iberian Mediterranean shore is more diverse and abundant than that of similar areas in the Atlantic. This region also has a greater degree of species endemism (seven Iberian pycnogonid species are endemic to the Mediterranean Sea), a pattern also found in other invertebrate and fish taxa [7,43]. This local species diversity differences may be due to the greater habitat diversity found in the Mediterranean region [44,45], mainly in the depth range 0–200 m, in contrast to the lower habitat heterogeneity found off the Spanish and Portuguese coasts [45]. This higher Mediterranean diversity observed may be also explained as Mediterranean marine biota includes both components of the endemic fauna typical from an enclosed sea and the fauna primarily derived from the Atlantic Ocean [4,44], as occurs with the Iberian Mediterranean pycnogonids (Table 2).

Two lessepsian pycnogonid species have been found in the Mediterranean basins, *Ammothea hilgendorfi* and *Nymphonella tapetis* [11,46]. The former species is restricted to the eastern basin and not yet recorded in the Iberian waters. The latter has been occasionally cited in the western basin nearby Barcelona. Nevertheless, lessepsian pycnogonids are few compared to other groups [6], possibly owing to their lack of a planktonic stage.

North-east Atlantic pycnogonids have been intensively studied with a recent synopsis of this fauna providing a total of 84 species [39]. More than a half of them (47 species) have been found in the Iberian Atlantic side excluding waters from the Strait of Gibraltar. Further shallow water surveys on the Atlantic coastline may extend the ranges of species belonging to the genus *Pycnogonum*, (e.g. *P*. *pusillum*, *P*. *nodulosum* and *P*. *plumipes*) which are currently only known from very shallow waters off the Mediterranean coast. Similarly, further collections from the Mediterranean continental shelf could expand the distribution of the common Atlantic *P*. *litorale* and deep-water Mediterranean surveys may extend the distribution of genera currently exclusive to the Atlantic (e.g. *Colossendeis*, *Cilunculus*, *Hedgpethia*, *Pantopipetta* and *Austrodecus*), which are commonly found at great depths. Although *Nymphon* is not exclusive to the Atlantic, more than 80% of its abundance is recorded in the Atlantic side. Only three out of 21 species displaying the general NE Atlantic distribution pattern (Fig. 5C) are also found in the Gibraltar area, indicating that many species have their distribution limit there, possible due to bathymetric and hydrographic constraints.

The pycnogonids from the western basin of Mediterranean Sea have been more intensively studied than those from the eastern basin [36,38,41]. To date, a total of 55 species have been recorded in the Mediterranean [15,38] with 14 species being endemic. Approximately 65% of the Mediterranean species have been also found in Iberian and Balearic Mediterranean waters (i.e. 36 out of 55 species). Twenty-nine Iberian species are distributed across both Mediterranean basins [41,47] and 12 are also found in the Balearic Sea (Table 2). There is one genus (*Neotrygaeus*) and seven species endemic to the Mediterranean Sea (Table 2).

The Strait of Gibraltar together with the Alboran Sea are considered to be biodiversity hotspots and key biogeographic and ecological areas [1,6,48]. These seem to be confluent zones of converging waters [49], and therefore they are considered as important biogeographical areas when comparing the faunal composition of the Atlantic and the Mediterranean [1]. Of the 27 pycnogonid species recorded within the Strait of Gibraltar, three are found in the Mediterranean but not found in the Atlantic region (*Ammothella biunguiculata*, *A*. *uniunguiculata* and *Rhynchothorax mediterraneus*) while another three species are found in the Atlantic but not in the Mediterranean (*Anoplodactylus arnaudae*, *Ascorhynchus pudicus* and *Hedgpethia atlantica*). Some researchers suggest that these areas act as buffer zones between the two major water masses [1] as contain an increased number of species, incorporating fauna from both abutting systems [1,5]. This is true for the pycnogonid fauna, with 24 species recorded in the adjacent Alboran Sea and 27 species in the Strait of Gibraltar with 3 being endemic. Moreover, the SAC anaysis (Fig. 4) provides an indication that the Alboran Sea is the second main region after the Catalan coast in terms of species numbers, and follows the pattern of the mean Mediterranean curve. The SAC of the Strait of Gibraltar has a slightly reduced slope compared to the Alboran Sea, and follows the pattern of the mean Atlantic region, suggesting a greater influence of the Atlantic fauna.

### Bathymetric distributions

Globally, fauna from the upper continental shelf has been far more intensively studied than deep-sea fauna. Our knowledge of Iberian benthic deep-sea fauna is rather incomplete [6] as only few surveys have been carried out [17,26,28,50,51] and some of them remained unpublished until now (e.g. DIVA-Artabria, INSUB). Most of the studies summarized in this review have been conducted in the upper continental shelf (0–100 m), with only 52 out of the 343 samplings located at depths below 1000 m.

Benthic abundance and diversity decrease drastically with depth [7,52]. These patterns are reflected in the Iberian pycnogonid fauna data, in both abundance (only the 2.5% of the records occurred beneath the 200 m), and the species diversity (31 exclusive continental shelf species versus 20 exclusive deep-sea species). The open Atlantic area, which is characterized mainly by deep-sea stations, has the poorest diversity and abundance compared to the other Atlantic sublittoral areas, such as the Portuguese coast (Fig. 4).

Fourteen out of the 19 species exclusive to the Iberian Atlantic region are present at depths equal or greater than 1000 m. In contrast, none of the Mediterranean deep pycnogonid species are exclusive to this region. This supports the hypothesis that Mediterranean deep-sea fauna originated from the richer Atlantic deep-sea fauna [7].

The bathymetric ranges described for the Iberian pycnogonid species are generally in accordance with their worldwide bathymetric limits (Table 2) [8,53]. The most abundant, but species poor genera (e.g. *Achelia* or *Ammothella*) were limited to locations near the coast (Fig. 2B), which may be not related to sampling bias but to depth limitations as they are confined to shallow waters globally. Some genera are highly eurybathic not only in the Iberian waters but also globally being the Iberian *Nymphon* the most highly diverse and eurybathic genus.

*Colossendeis* is a cold-water genus distributed worldwide but is far more abundant in Arctic and Antarctic waters [54]. In these cold-water areas it can be found at very shallow depths [34]. It has been well diversified in cold, deep waters (including bipolar distribution), suggesting that it has a long evolutionary history in cold, well-oxygenated waters [55]. The Iberian *Colossendeis* are restricted at depths greater than 900 m and found only in the Atlantic side. The higher water temperatures of the Mediterranean and the contrary bottom-current of the Gibraltar sill acting as a biogeographic barrier [7,28] may account for their absence in the Mediterranean Sea.

Given that deep-sea assemblages of Mediterranean pycnogonid fauna are still poorly known due to limited sampling efforts (only one of the 87 Mediterranean sampling locations is deeper than 1000 m), the depletion observed of the Mediterranean species accumulation curve may be mostly representative of the shallow fauna. Further deep water surveys throughout the Mediterranean Sea should therefore be prioritized in order to conclusively exclude the presence of any Atlantic species.

## Conclusions

A total of 17762 specimens belonging to 65 Iberian species of pycnogonids have been recorded to the date. The family Acheliidae is by far the most abundant (80% of specimens collected). Although decent sampling efforts have been conducted throughout the Iberian waters, the SAC analysis indicates that further sampling would likely increase the number of pycnogonid species found in this region. While the total number of species is greater in the Iberian Atlantic side, Iberian Mediterranean waters are richer than the Atlantic, with the Strait of Gibraltar and the Alboran Sea being biodiversity hot spots and act as buffer zones across the Atlantic-Mediterranean confluence. A sampling bias is evident regarding the bathymetric cell grids analyzed, as only the 15% of the sampling sites were at depths greater than 1000 m. As a priority, further sampling should be carried out mainly on the deep Iberian Mediterranean side.

S1 Table contains references and new data by species and zone. References are codified following the manuscript citation numbers (from 56 to 72) and are listed in reference list.

## Supporting Information

S1 Information Discussion on the effects of grid cell size. (DOCX)

S1 Table References and new data by species and zone. New data included the following surveys: Dragoneres (Balearic Islands, N = 63), DIVA-Artabria (N = 72), the Museo Bocage collection (Portugal, N = 167), El Cachucho (Le Danois Bank, N = 12) and INSUB (N = 83). Data derived from the PhD of Munilla [12] (N = 8508) and Soler-Membrives [15] (N = 3182) not published elsewhere are also considered new data. References [56–72] are also included in the reference list.
(DOCX)

S2 Table Within region characterization of Iberian areas used for the analysis. Latitudinal and longitudinal limits of each area are delimited, and main factor of influence are provided.
(DOCX)
