# Supplementary material for: PYCNOIB: Biodiversity and Biogeography of Iberian Pycnogonids
Source: PLoS One. 2015 Mar 17;10(3):e0120818. doi: 10.1371/journal.pone.0120818 (PMC4363526; doi:10.1371/journal.pone.0120818)
Supplement: S1 Information — (DOCX) [file pone.0120818.s001.docx]

**Supporting information**. Discussion on the effects of grid cell size.

The grid cell size selected was 0.3 degree of latitude by 0.3 degree of longitude. Choosing an appropriate cell size is not always simple. Accuracy or resolution typically refers to the size of the grid cell. One can select the grid cell size that shows the maximum information content in the plotted map. Nonetheless, the complexity increases the smaller the grid cell size. Many times, choosing a smaller cell size does not necessarily mean more accurate results, until a threshold value, when the accuracy then declines. The optimal grid cell size is the one where further refinement does not change the accuracy of the results. It must be balanced the application's need for spatial resolution (higher resolution) with practical requirements for better display and interpretation of data (lower resolution). Choice of cell size is a function of spatial variability of the feature in question and the purpose/objective of the GIS.

In that case, cell size of 0.3° of latitude by 0.3° of longitude was selected to get a sufficient spatial resolution but a proper display of the represented patterns. This resolution gives an acceptable compromise for spatial accuracy.

In order to obtain a comparison of results using different size of grid cells and to check the effect of the grid cell size, different scenarios have been assessed. Three different cell sizes were selected: 0.1 by 0.1 degrees, 0.3 by 0.3 degrees and 0.5 by 0.5 degrees to plot the number of species and the number of sampling sites. Cell sizes should be properly selected to adequately reflect spatial variations. When the cell size increases the quality of the plot results decreases. The next figure shows the number of sampling sites (1) and the number of species (2) at different grid cell sizes: 0.1º by 0.1º (A), 0.3º by 0.3º (B) and 0.5º by 0.5º (C), respectively.


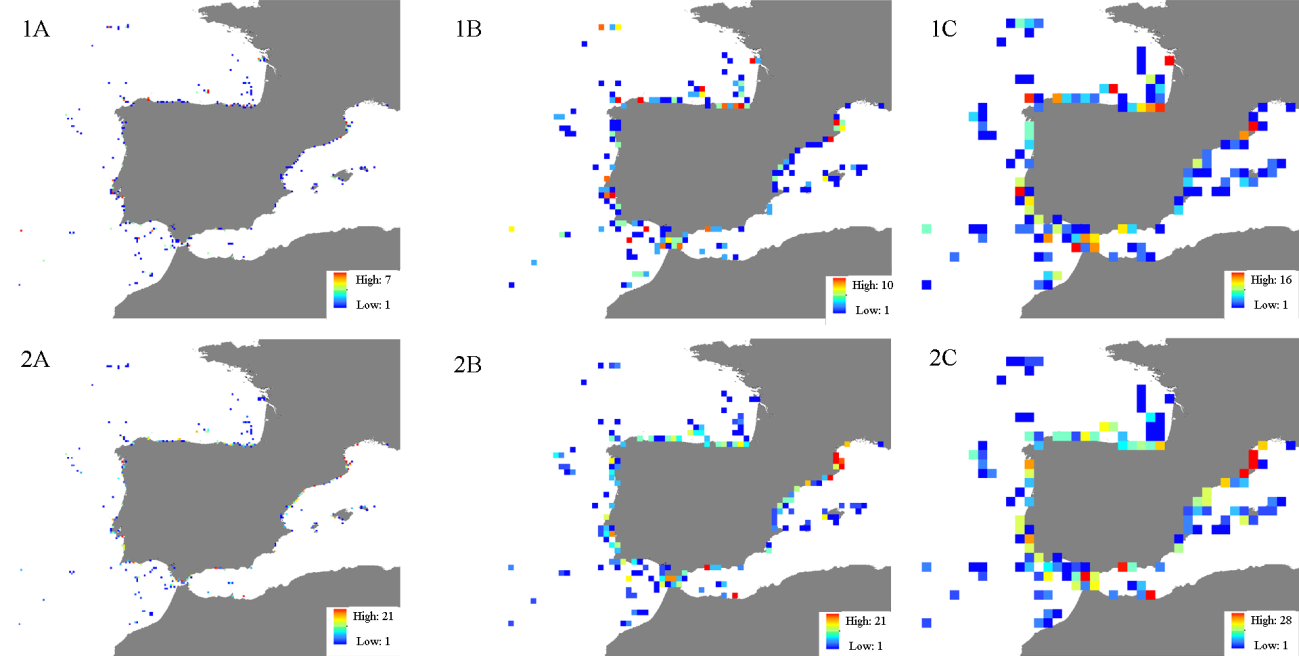


The 0.1 by 0.1 degree grid cell size maps plot more detailed distributions but giving no more accuracy and getting harder the interpretation of the results. The 0.5 by 0.5 degree grid cell size maps still show the main biogeographic patterns, but some specific details may be diluted. The 0.3 by 0.3 degree grid cell size show an adequate accuracy for both represented plots (number of sampling sites and number of species) and is displayed in a size easier to interpret spatial variations.

The main patterns found are not likely to be misinterpreted by the selection of a higher/smaller size of the grid cell than needed. In this case, two alternative scenarios could be considered: a) a smaller cell size than the selected one (e.g. 0.1 by 0.1 degree); this should not have any effect to the interpretation of the results but a much finer visualization of the results – in our point of view, not necessarily a better visualization; b) a bigger cell size than the selected one (e.g. 0.5 by 0.5 degrees); a bigger cell size could drop to a decrease in the accuracy of the results, and therefore a misinterpretation of the distribution patterns – e.g. exclusive species would seem to have a broader distribution than they have, localized areas very poorly or highly sampled could become faded, localized hotspots of species diversity could become blurred and go unnoticed.
