# Supplementary material for: PYCNOIB: Biodiversity and Biogeography of Iberian Pycnogonids
Source: PLoS One. 2015 Mar 17;10(3):e0120818. doi: 10.1371/journal.pone.0120818 (PMC4363526; doi:10.1371/journal.pone.0120818)
Supplement: S1 Table — New data included the following surveys: Dragoneres (Balearic Islands, N = 63), DIVA-Artabria (N = 72), the Museo Bocage collection (Portugal, N = 167), El Cachucho (Le Danois Bank, N = 12) and INSUB (N = 83). Data derived from the PhD of Munilla [12] (N = 8508) and Soler-Membrives [15] (N = 3182) not published elsewhere are also considered new data. References [56–72] are also included in the reference list. (DOCX) [file pone.0120818.s002.docx]

| **Table S2.** References and new data by species and zone. New data included the following surveys: Dragoneres (Balearic Islands), DIVA-Artabria, the Museo Bocage collection (Portugal), El Cachucho (Le Danois Bank) and INSUB. Data derived from the PhD of Munilla [12] and Soler-Membrives [15] not published elsewhere are also considered new data.   \| **Species** \| **ATL** \| **MED** \| **GIB** \| \| --- \| --- \| --- \| --- \| \| *Achelia echinata* Hodge, 1864 \| [22,23,24,25,28,29,61,62,63]INSUB, Bocage \| [11,12,13,14,15,16,17,18,19,20,21,22,36,64,65,66,67,68]INSUB, Dragoneres \| [17,27,36,69]INSUB \| \| *Achelia langi* (Dohrn, 1881) \| [23,24,29]Bocage \| [11,12,13,14,16,18,21,68] \| [27] \| \| *Achelia vulgaris* (Costa, 1861) \| [23,24,25,63]Bocage \| [11,12,13,14,15,17,18,22,64,65,68] \| [17] \| \| *Ammothella appendiculata* (Dohrn, 1881) \|  \| [11,14,18,67,68] \|  \| \| *Ammothella biunguiculata* (Dohrn, 1881) \|  \| [12,13,68] \| [17] \| \| *Ammothella gibraltarensis* Munilla, 1993 \|  \|  \| [17] \| \| *Ammothella longioculata* (Faraggiana, 1940) \|  \| [18] \|  \| \| *Ammothella longipes* (Hodge, 1864) \| [22,23,24,25,61,62,63]INSUB, Bocage \| [11,12,14,15,16,18,19,20,21,64,65,66,67,68] \| [17,27,36,69] \| \| *Ammothella tubicen* Stock, 1978 \| [50] \|  \|  \| \| *Ammothella uniunguiculata* (Dohrn, 1881) \|  \| [11,14,15,16,18,64,66] \| [27] \| \| *Anoplodactylus angulatus* (Dohrn, 1881) \| [25,63]INSUB \| [11,12,13,14,15,17,18,22,68]INSUB \| [17,27] \| \| *Anoplodactylus arnaudae* Stock, 1978 \| [28,70] \|  \| [28] \| \| *Anoplodactylus nanus* Krapp, Kocak & Kagatan, 2008 \|  \| [15] \|  \| \| *Anoplodactylus oculatus* Carpenter, 1905 \| Bocage \|  \|  \| \| *Anoplodactylus petiolatus* (Krøyer, 1844) \| [22,23,24,25,26,28,29,50,62,63]INSUB, Bocage, DIVA-Artabria \| [11,18,19,20,65,68] \| [17,28] \| \| *Anoplodactylus pygmaeus* (Hodge, 1864) \| [24,25,29,63]Bocage \| [12,13,14,16,17,18,67,68] \| [27] \| \| *Anoplodactylus robustus* (Dohrn, 1881) \|  \| [17,68] \|  \| \| *Anoplodactylus typhlops* Sars, 1888 \| [28]Cacucho, DIVA-Artabria \|  \|  \| \| *Anoplodactylus virescens* (Hodge, 1864) \| [22,23,24,25,61,62,63]INSUB, Bocage \| [11,15,18,65,68] \| [27] \| \| *Ascorhynchus abyssi* Sars, 1877 \| [23] \|  \|  \| \| *Ascorhynchus castelli* (Dohrn, 1881) \| INSUB \| [20,68] \|  \| \| *Ascorhynchus pudicus* Stock, 1970 \| [70] \|  \| [28] \| \| *Ascorhynchus simile* Fage, 1942 \| [22]INSUB \| [14,68] \|  \| \| *Ascorhynchus turritus* Stock, 1978 \| [50] \|  \|  \| \| *Austrodecus conifer* Stock, 1991 \| [50] \|  \|  \| \| *Bathypallenopsis juttingae* (Stock, 1964) \| [26] \|  \|  \| \| *Bathypallenopsis longirostris* (Wilson, 1881) \| [26,71] \|  \|  \| \| *Bathypallenopsis scoparia* (Fage, 1956) \| [26] \| [22] \| [28] \| \| *Callipallene brevirostris* (Johnston, 1837) \| [23,24,29] \| [13,18,28] \|  \| \| *Callipallene emaciata* (Dohrn, 1881) \| [23,24,25,28,29,62]Bocage \| [11,12,15,16,18,22,64,65,68]Dragoneres \| [17,27,36] \| \| *Callipallene phantoma* (Dohrn, 1881) \| [28]Cachucho \| [12,13,18,20,22]INSUB \|  \| \| *Callipallene producta* (Sars, 1881) \| [28]Cachucho, DIVA-Artabria \| [12,16,17,18,19,20,28,68] \| [28] \| \| *Callipallene spectrum* (Dohrn, 1881) \| [29] \| [11,18,19,20,68]Dragoneres \| [17] \| \| *Callipallene tiberi* (Dohrn, 1881) \| [22,28,69]INSUB \| [16,18,19,65] \| [17,28]INSUB \| \| *Cilunculus alcicornis* Stock, 1978 \| [70]Cachucho \|  \|  \| \| *Cilunculus europaeus* Stock, 1978 \| [28,50,70]Cachucho \|  \|  \| \| *Colossendeis angusta* Sars, 1877 \| [28,71] \|  \|  \| \| *Colossendeis arcuata* Milne-Edwards, 1885 \| [28,51] \|  \|  \| \| *Colossendeis clavata,* Meinert, 1899 \| [51,71] \|  \|  \| \| *Colossendeis colossea* Wilson, 1881 \| [23,24,28,51,70,71] \|  \|  \| \| *Colossendeis macerrima* Wilson, 1881 \| [28,51,70,71] \|  \|  \| \| *Endeis charybdaea* (Dohrn, 1881) \| [69] \| [68] \|  \| \| *Endeis spinosa* (Montagu, 18808) \| [23,24,25,29,62,63,69]INSUB, Bocage \| [11,14,15,18,19,20,22,65,68] \| [17,27,36] \| \| *Hannonia stocki* Munilla, 1993 \|  \|  \| [17] \| \| *Hedgpethia atlantica* (Stock, 1970) \| [70] \|  \| [28] \| \| *Nymphon caldarium* Stock, 1987 \|  \|  \| [28] \| \| *Nymphon gracile* Leach, 1814 \| [22,23,24,29,62,69,72]Bocage, Cachucho \| [11,12,13,14,64,68] \| [27] \| \| *Nymphon laterospinum* Stock, 1963 \| [50] \|  \|  \| \| *Nymphon macrum* Wilson, 1880 \| [23,24] \|  \|  \| \| *Nymphon puellula* Krapp, 1973 \|  \|  \| INSUB \| \| *Nymphon tricuspidatus* Soler-Membrives & Munilla, 2011 \| Cachucho, DIVA-Artabria \|  \|  \| \| *Nymphon tubiferum* Stock, 1978 \| [50] \|  \|  \| \| *Nymphonella tapetis* Ohshima, 1927 \|  \| [11,65,68] \|  \| \| *Pantopipetta armoricana* Stock, 1978 \| [50,70]DIVA-Artabria \|  \|  \| \| *Paranymphon spinosum* Caullery, 1896 \| [23,24,26,28,50]INSUB, DIVA-Artabria \|  \| [68] \| \| *Pentapycnon geayi* Bouvier, 1911 \|  \|  \| [17] \| \| *Pycnogonum litorale* (Strom, 1762) \|  \| [16,68] \|  \| \| *Pycnogonum nodulosum* Dohrn, 1881 \| [23,24,26,69]Cachucho \|  \|  \| \| *Pycnogonum plumipes* Stock, 1960 \|  \| [11,12,13,14,15,17,19,20,22,68] \|  \| \| *Pycnogonum pusillum* Dohrn, 1881 \|  \| [68] \|  \| \| *Rhynchothorax mediterraneus* Costa, 1861 \|  \| [22] \| [28] \| \| *Rhynchothorax voxorinus* Stock, 1966 \|  \| [68] \|  \| \| *Tanystylum conirostre* (Dohrn, 1881) \| [22,23,24,63]INSUB, Bocage \| [14,16,18,19,20,22,64,65,66,67,69]Dragoneres \| [17,27,36]INSUB \| \| *Tanystylum orbiculare* Wilson, 1878 \| [23,24] \| [11,14,15,18,20,21,64,68] \|  \| \| *Neotrygaeus communis* Dohrn, 1881 \|  \| [11,18,21,22,64,68] \|  \| |
| --- | --- | --- | --- | --- | --- | --- | --- | --- | --- | --- | --- | --- | --- | --- | --- | --- | --- | --- | --- | --- | --- | --- | --- | --- | --- | --- | --- | --- | --- | --- | --- | --- | --- | --- | --- | --- | --- | --- | --- | --- | --- | --- | --- | --- | --- | --- | --- | --- | --- | --- | --- | --- | --- | --- | --- | --- | --- | --- | --- | --- | --- | --- | --- | --- | --- | --- | --- | --- | --- | --- | --- | --- | --- | --- | --- | --- | --- | --- | --- | --- | --- | --- | --- | --- | --- | --- | --- | --- | --- | --- | --- | --- | --- | --- | --- | --- | --- | --- | --- | --- | --- | --- | --- | --- | --- | --- | --- | --- | --- | --- | --- | --- | --- | --- | --- | --- | --- | --- | --- | --- | --- | --- | --- | --- | --- | --- | --- | --- | --- | --- | --- | --- | --- | --- | --- | --- | --- | --- | --- | --- | --- | --- | --- | --- | --- | --- | --- | --- | --- | --- | --- | --- | --- | --- | --- | --- | --- | --- | --- | --- | --- | --- | --- | --- | --- | --- | --- | --- | --- | --- | --- | --- | --- | --- | --- | --- | --- | --- | --- | --- | --- | --- | --- | --- | --- | --- | --- | --- | --- | --- | --- | --- | --- | --- | --- | --- | --- | --- | --- | --- | --- | --- | --- | --- | --- | --- | --- | --- | --- | --- | --- | --- | --- | --- | --- | --- | --- | --- | --- | --- | --- | --- | --- | --- | --- | --- | --- | --- | --- | --- | --- | --- | --- | --- | --- | --- | --- | --- | --- | --- | --- | --- | --- | --- | --- | --- | --- | --- | --- | --- | --- | --- | --- | --- | --- | --- | --- | --- | --- | --- | --- | --- | --- | --- |
