# Supplementary material for: PYCNOIB: Biodiversity and Biogeography of Iberian Pycnogonids
Source: PLoS One. 2015 Mar 17;10(3):e0120818. doi: 10.1371/journal.pone.0120818 (PMC4363526; doi:10.1371/journal.pone.0120818)
Supplement: S2 Table — Latitudinal and longitudinal limits of each area are delimited, and main factor of influence are provided. (DOCX) [file pone.0120818.s003.docx]

**Table S1**. Within region characterization of Iberian areas used for the analysis. Latitudinal and longitudinal limits of each area are delimited, and main factor of influence are provided.

| **Area** | **Code** | **Region** | **Latitudinal limits** | **Longitudinal limits** | **Main factor of influence** | **Reference** |
| --- | --- | --- | --- | --- | --- | --- |
| Bay of Biscay | BIS | Atlantic | Coast line (43ºN) - 46.5ºN | Coast line (1ºE) - 5ºW | General weak oceanic circulation, persistent slope current and frequent eddies | [56] |
| North Iberian Peninsula | NIB | Atlantic | Coast line (43ºN) - 46.5ºN | 5ºW - 7ºW |  |  |
| Galician coast | GAL | Atlantic | 42ºN - 46.5ºN | 7ºW - 9ºW | Very exposed to the Eastern North Atlantic current | [57] |
| Portugal | POR | Atlantic | 36ºN - 42ºN | Coast line (6.5ºW) - 10ºW | Iberian Poleward current | [58] |
| Open Atlantic ocean | OATL | Atlantic | 44ºN - 47ºN | 9ºW - 15ºW | Open waters |  |
|  |  |  | 33ºN - 35ºN | 7ºW - 15ºW |  |  |
| Strait of Gibraltar | GIB | Gibraltar | 35ºN - 37ºN | 6.5ºW - Estepona (5ºW) | Mediterranean waters at depth westward and Atlantic waters above eastward | [1] |
| Alboran Sea | ALB | Mediterranean | 35ºN - 37ºN | 4.5ºW - 2ºW | Atlantic Jet and Northern Current from the Catalan Sea, and Eastern and Western Alboran Gyres | [1] |
| Eastern Iberian Mediterranean | EMED | Mediterranean | 37ºN - 41ºN | 1ºW - Ebro Delta (0.7ºE) |  |  |
| Catalan coast | CAT | Mediterranean | Ebro Delta (40.7ºN) - Boundary (43ºN) | Ebro Delta (0.7ºE) - Boundary (3.6ºE) | Northern Current of the Catalan Sea from the Gulf of Lion | [59] |
| Balearic Islands | BAL | Mediterranean | 38ºN - 40ºN | 1ºE - 5ºE | Islands, Balearic current, Balearic channels | [60] |
|  |  |  |  |  |  |  |
|  | | | | |  |  |
|  | | | | | | |
|  | | | | | | |
|  | | | | | | |
|  | | | | | |  |
